# The first two whole mitochondrial genomes for the genus *Dactylis* species: assembly and comparative genomics analysis

**DOI:** 10.1186/s12864-024-10145-0

**Published:** 2024-03-04

**Authors:** Guangyan Feng, Yongjuan Jiao, Huizhen Ma, Haoyang Bian, Gang Nie, Linkai Huang, Zheni Xie, Qifan Ran, Wenwen Fan, Wei He, Xinquan Zhang

**Affiliations:** 1 College of Grassland Science and Technology, Sichuan Agricultural University, Chengdu, 611130 China; 2 Grassland Research Institute, Chongqing Academy of Animal Science, Chongqing, 402460 China

**Keywords:** Mitochondrial genome, RNA editing sites, Repetitive DNA, Comparative analysis, DNA transfer, Phylogeny

## Abstract

**Background:**

Orchardgrass (*Dactylis glomerata* L.), a perennial forage, has the advantages of rich leaves, high yield, and good quality and is one of the most significant forage for grassland animal husbandry and ecological management in southwest China. Mitochondrial (mt) genome is one of the major genetic systems in plants. Studying the mt genome of the genus *Dactylis* could provide more genetic information in addition to the nuclear genome project of the genus.

**Results:**

In this study, we sequenced and assembled two mitochondrial genomes of *Dactylis* species of *D. glomerata* (597, 281 bp) and *D. aschersoniana* (613, 769 bp), based on a combination of PacBio and Illumina. The gene content in the mitochondrial genome of *D. aschersoniana* is almost identical to the mitochondrial genome of *D. glomerata*, which contains 22–23 protein-coding genes (PCGs), 8 ribosomal RNAs (rRNAs) and 30 transfer RNAs (tRNAs), while *D. glomerata* lacks the gene encoding the Ribosomal protein (*rps1*) and *D. aschersoniana* contains one pseudo gene (*atp8*)*.* Twenty-three introns were found among eight of the 30 protein-coding genes, and introns of three genes (*nad 1*, *nad2*, and *nad5*) were trans-spliced in *Dactylis aschersoniana.* Further, our mitochondrial genome characteristics investigation of the genus *Dactylis* included codon usage, sequences repeats, RNA editing and selective pressure. The results showed that a large number of short repetitive sequences existed in the mitochondrial genome of *D. aschersoniana*, the size variation of two mitochondrial genomes is due largely to the presence of a large number of short repetitive sequences. We also identified 52–53 large fragments that were transferred from the chloroplast genome to the mitochondrial genome, and found that the similarity was more than 70%. ML and BI methods used in phylogenetic analysis revealed that the evolutionary status of the genus *Dactylis.*

**Conclusions:**

Thus, this study reveals the significant rearrangements in the mt genomes of Pooideae species. The sequenced *Dactylis* mt genome can provide more genetic information and improve our evolutionary understanding of the mt genomes of gramineous plants.

**Supplementary Information:**

The online version contains supplementary material available at 10.1186/s12864-024-10145-0.

## Background

Orchardgrass (*Dactylis glomerata* L), belonging to the angiosperm family Gramineae, is widely distributed across Europe, temperate and tropical Asia, North Africa, and the Canary Islands [1]. It is widely used for forage, grazing, and hay modulation due to its high adaptability, nutritional value, and biomass production [2]. *Dactylis* has a history of more than 200 years of cultivation in North America, where it is planted in large areas and serves as one of the major forge resources. For over 100 years, orchardgrass has been essential for herbage-based livestock production in temperate regions worldwide. Studies over the past decades focused on molecular marker-assisted breeding and important agronomic traits, with few studies investigating the molecular basis for genetic breeding, phylogenetics, and germplasm identification, which can affect the conservation and development of species. Thus, further studies are necessary to clarify the classification and taxonomic relationships of species of the genus *Dactylis* L and develop more effective methods for conserving and utilizing these species.

Mitochondria (mt) originated 1.5 billion years ago from the endosymbiotic integration of an a-proteobacterium and are key organelles ubiquitous in all eukaryotic cells [3]. As the center of cellular energy metabolism, mt plays an important role in plant development and productivity [4, 5]. The chloroplast (cp) and mt genomes differ from the nuclear genome in that cp and mt exhibit maternal inheritance. Unlike the cp genome, the mt genome lacks a conserved gene order, which further reflects the complexity of plant mt genomes [6, 7]. Recent studies reported that the processes involved in mt genome evolution differ significantly among major eukaryotic populations, including fungi, plants, and animals [8]. Unlike animals and fungi, plant mt genomes vary greatly in genome size, structure, gene content, and recombination rate [9–12]. For example, in flowering plants, the size of the mt genome varies significantly even within the same species, with genome size ranging from 200 to 2400 kb [13–15]. The size variation may be due to non-coding DNA, such as repetitive DNA sequences, introns, and the migration of exogenous DNA from cp and nuclear genomes into the plant mt genomes [16].

In addition, unlike the conserved structure of plant cp genome, structural variation is ubiquitous in plant mt genomes, even among members of the same species, mainly due to the presence of repetitive sequences [17, 18]. Repetitive sequences can be classified into simple sequence repeats (SSRs), tandem repeats, and dispersed repeats. These repeats play an important role in the formation of plant mt genome structure through genome rearrangement, genome sequence replication, inversion, insertion, and deletion [19]. Repetitive sequences are the source of recombination in the genome and trigger various dynamic changes in mt genome structure and evolution [20]. To date, homologous recombination of the mt genome involving repetitive sequences has been studied in many plant species [10, 21, 22], and many repetitive sequences frequently undergo intramolecular recombination, making the sequencing of plant mt genomes, especially angiosperms, very difficult [23]. Thus, mt genomes are valuable sources of genetic information for phylogenetic studies and the investigation of essential cellular processes. Plant mt genome exhibits several features (large size, low conservation rate across species, and high structural divergence), making its complete assembly challenging. Recent advances in next-generation sequencing technologies combining short-read and long-read sequencing technologies from Illumina and PacBio have made the analysis of large genomes easy [24]. The PacBio platform overcomes the read length deficiency of the Illumina platform (which cannot span large repetitions), thus improving the coverage and assembly accuracy of the unassembled genomic regions and greatly promoting the study of plant mt genomes. Consequently, the number of plant mt genomes deposited into the NCBI Plant Organelle Genome Database continues to increase, from lower algae to higher angiosperms. However, the mt genomic data of many plant families are yet to be reported. Therefore, it is necessary to further investigate the mt genomic information to resolve the phylogenetic relationships among more plant species.

In our previous studies, the *Dactylis* genome and the cp genomes of 14 *Dactylis* species were reported, and the phylogenetic relationship between species of genus *Dactylis* L was established [25]. Such studies can provide a reference for further exploring the molecular genetics and phylogenetics of Gramineae grasses. In this study, to investigate the genetic information and evolutionary status of the genus *Dactylis* L, we assembled and annotated the first mt genome of *Dactylis*, and compared it with published mt genomes of the subfamily Pooideae. The main objectives were: (1) to investigate the composition, genome size, repetitive sequences, codon bias, and RNA editing sites of the mt genomes of the genus *Dactylis* L; (2) to explore the gene transfer between the cp genome and the mt genome of the genus *Dactylis* L; (3) to estimate the phylogenetic relationship between the genus *Dactylis* L and other Gramineae members using mt genomic data. The results of this study could provide insights to further understand the evolution and phylogeny of the Gramineae plants.

## Results

### Library quality assessment and sequencing data evaluation

We first evaluated the quality of the original sequencing data of the samples and plotted the base content distribution map. According to the principle of base complementation, the content of AT and CG should be equal, and the content of N reflects the sequencing quality. The smaller the proportion of N, the higher the sequencing quality. The read length distribution of the third-generation sequencing showed that the contents of A, T, C, and G bases were equal and stabilized in a straight line (Fig. S1), indicating that the sequencing quality was good. The distribution statistics of the basic error rate can show the library quality. The sequencing error rate was about 0.02% (Fig. S2), and the base quality of the library was sufficient for subsequent analysis. The sequencing data of *D. aschersoniana* was about 8.3G, with Q20 of 97.18%, Q30 of 91.91%, and the GC of 43.69%. The data volume of *D. glomerata* was about 8.5 G, with Q20 of 97.64%, Q30 of 92.91%, and GC content of 43.79%. These indicated that the quality of mt genomes of two *Dactylis* species was high, and the sequencing data can be used for further analysis (Tables S1 and S2). In addition, we evaluated the sequencing depth of coverage in the chloroplast and mitochondrial genomes of the *Dactylis* genus. The maximum sequencing depth of coverage for the *Dactylis glomerata* mitochondrial genome was 3535 × , with an average of 90.45 × . The *Dactylis glomerata* chloroplast genome had a maximum sequencing depth of coverage of 1986 × , with an average of 1600.27 × . The sequencing depth of coverage for the mitochondrial genome of *Dactylis aschersoniana* was 1816 × maximum and 54.99 × average, while for the chloroplast genome it was 996 × maximum and 796.09 × average (Fig. S3).

### Chloroplast and mitochondrial genome organization

**Fig. 1:**
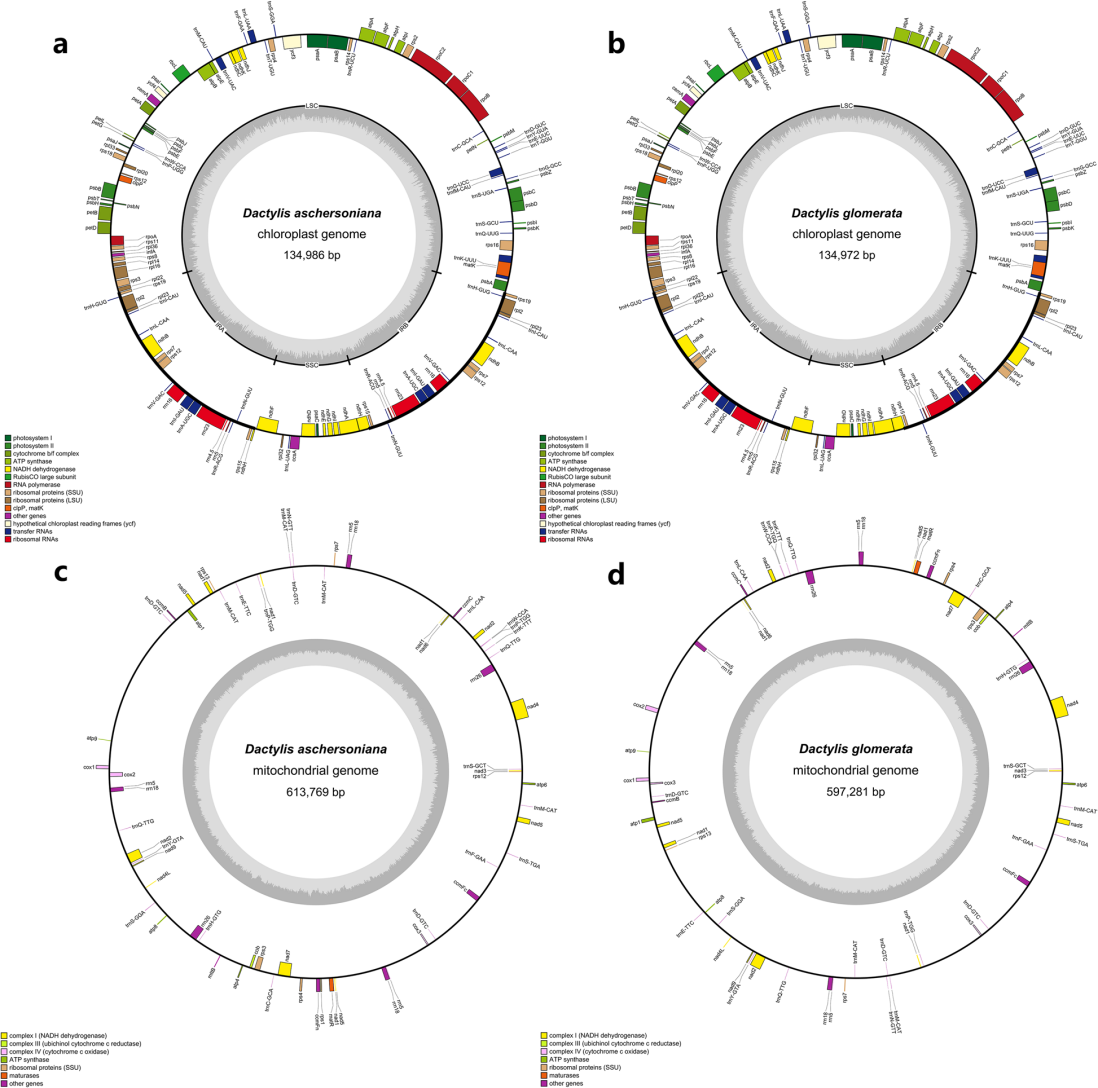
Annotated chloroplast and mitochondrial genomes of *Dactylis aschersoniana* and *Dactylis glomera*. **a** and **b** Gene map showing annotated genes with different functional groups inside and outside the circle transcribed clockwise (outside) and counterclockwise (inside). The dark gray plot in the inner circle indicates the GC content. **c** and **d** Gene map showing annotated genes with different functional groups, which are color-coded on the outer circle transcribed clockwise (outside) and counterclockwise (inside). The dark gray plot in the inner circle shows the GC content

We assembled and annotated the complete cp and mt genomes of *D. aschersoniana* and *D. glomerata*. The cp and mt genomes of the two *Dactylis* species exhibited a circular structure. The cp genome size ranged from 134, 972 bp to 134, 986 bp, and the mt genome size ranged from 587, 289 bp (*D. glomerata*) to 613, 769 bp (*D. aschersoniana*), which was about 4.5 times that of the cp genome (Fig. 1). The GC content of the mt genome was 44.01% and 44.08% for *D. glomerata* and *D. aschersoniana*, respectively (Table S3). The base contents of the mt genomes were T (27.98%-28.14%), A (27.85%-27.94%), C (21.98%-22.03%), and G (21.98%-22.11%) (Table 1). The non-coding sequences ranged from 530, 330 bp (*D. glomerata*) to 546, 889 bp (*D. aschersoniana*), representing 88.79%-89.10% of the total genome (Table 1). In addition, we also identified protein-coding genes, tRNA, rRNA, and introns. The GC content of the protein-coding genes was much lower than in other regions (Table 1). In addition, comparative analysis of mVISTA was performed in two mitochondrial genomes of *D. glomerata* and *D. aschersoniana*The results showed that the majority of variants resided in the intergenic region, including *nad1*-*rps7*, *ccmB*-*atp9*, *cox1*-*cox2*, *mttB*-*atp4*, *rrn5*-*cox3* (Fig. S4).

**Table 1:** Structural features of the mitochondrial genomes of the two *Dactylis* species

Feature	A (%)	T (%)	G (%)	C (%)	GC (%)	Size (bp)	Proportion in the genome (%)
Dactylis aschersoniana							
genome	27.85	28.14	21.98	22.03	44.01	613769	100
CDS	26.29	31.03	21.55	21.14	42.69	28350	4.62
Cis-spliced intron	24.87	22.54	27.73	24.87	52.6	23545	3.84
tRNA	22.12	26.13	29.23	22.52	51.75	1745	0.28
rRNA	25.59	21.22	29.71	23.47	53.19	13240	2.16
Non-coding region	28.16	28.38	21.74	21.72	43.46	546889	89.10
Dactylis glomerata							
genome	27.94	27.98	22.11	21.98	44.08	597281	100
CDS	26.01	31.23	21.55	21.21	42.76	28515	4.77
Cis-spliced intron	24.85	22.53	27.73	24.88	52.61	23525	3.94
tRNA	22.08	26.39	29.2	22.32	51.53	1671	0.28
rRNA	25.59	21.22	29.71	23.47	53.19	13240	2.22
Non-coding region	28.26	28.21	21.87	21.66	43.53	530330	88.79

### Gene composition of the mitochondrial genomes

As shown in Table 2, 61 unique genes were present in the mt genomes of the two *Dactylis* species, among which 22–23 genes were protein-coding genes, eight genes were rRNAs, 30 genes were tRNAs, and one was a pseudogene. *Atp8* existed as a pseudogene in the mt genome of *D. glomerata* (Table 2). Moreover, three genes (*rrn26*, *trnP-TGG*, and *trnQ-TTG*) were present in two copies, while four genes (*rrn5*, *rrn18*, *rrn26*, and *trnD-GTC*) were present in three copies and one gene (*trnM-CAT*) were present in multiple copies (three and four copies) in the two mt genomes. Three copies of *trnM-CAT* were found in *D. glomerata*, and four copies of *trnM-CAT* were found only in *D. aschersoniana*. Additionally, two copies of the *cox3* gene were found only in *D. glomerata*. Eight protein-coding genes (PCGs) contained introns, among which two (*ccmFc* and *rps3*) contained a single intron, one (*cox2*) contained two introns, one (*nad4*) contained three introns, and four (*nad1*, *nad2*, *nad5*, and *nad7*) contained four introns. Three genes (*nad1*, *nad2*, and *nad5*) were trans-spliced, while two genes (*nad 4* and *nad7*) were cis-spliced in *Dactylis aschersoniana*.

**Table 2:** Gene composition of the mitochondrial genomes of *Dactylis aschersoniana* and *Dactylis glomerata*

Gene group	Gene name
ATP synthase	^#^atp8ª atp1 atp4 atp6 atp8 atp9
Cytochrome c biogenesis	ccmB ccmC ccmFc^*^ ccmFn
Ubiquinol cytochrome c reductase	cob
Cytochrome c oxidase	cox1 cox2^**^ cox3(2)ª cox3^^^
Maturase	matR
Transport membrane protein	mttB
NADH dehydrogenase	nad1^****^ nad2^****^ nad3 nad4^***^ nad4L nad5^****^ nad6 nad7^****^ nad9
Ribosomal proteins (LSU)	
Ribosomal proteins (SSU)	rps1^^^ rps12 rps13 rps3^*^ rps4 rps7
Succinate dehydrogenase	
Ribosomal RNAs	rrn18(3) rrn26(2) rrn5(3)
Transfer RNAs	trnC-GCA trnD-GTC(3) trnE-TTC trnF-GAA trnH-GTG trnK-TTT trnL-CAA trnM-CAT(3)^a^ trnM-CAT(4)^^^ trnN-GTT trnP-TGG(2) trnQ-TTG(2) trnS-GCT trnS-GGA trnS-TGA trnW-CCA trnY-GTA

The introns of *nad1*, *nad2*, and *nad5* were trans-spliced in *Dactylis aschersoniana*

^*^intron number, * one intron; ** two introns; *** three introns; **** four introns

^#^Gene, pseudo gene exist in *Dactylis glomerata*; Gene (), copy numbers of the multi-copy genes

^^^present only in *Dactylis aschersoniana*

ªpresent exist in *Dactylis glomerata*

### Condon usage analysis of the PCGs

**Fig. 2:**
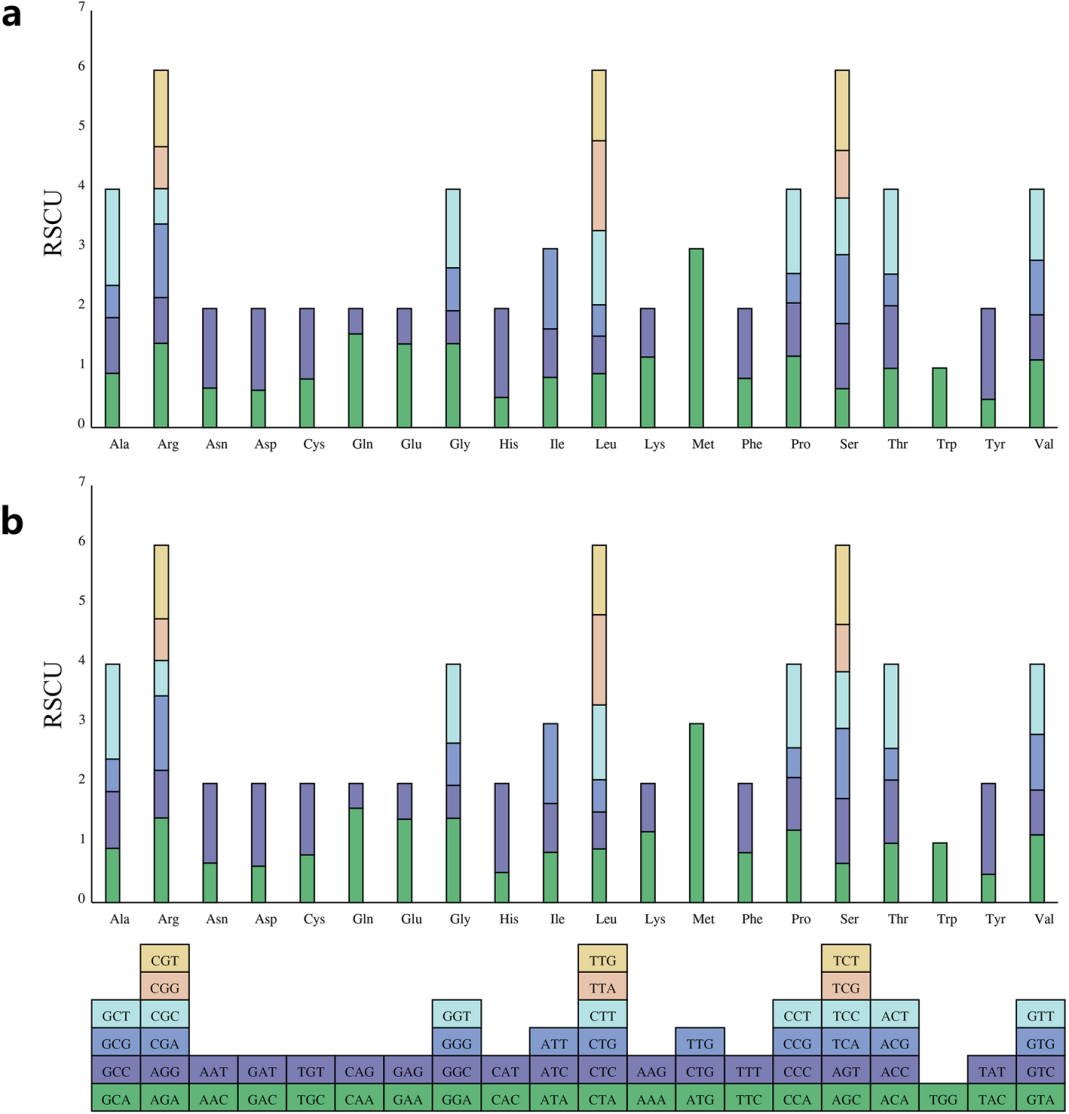
Codon bias analysis of the mitochondrial (mt) genome*.* **a** *Dactylis glomerata*. **b** *Dactylis aschersoniana*

The combined effects of natural selection, drift, and gene mutation during the long-term evolution of plants led to the differences in the codon usage frequency of most plants. The calculations for the codon usage of the PCGs within the *D. aschersoniana* and *D. glomerata* mt genomes are summarized in Table S4. Most PCGs presented an ATG as the start codon, while ACGs were the start codon in the *nad1* and *nad4L* genes. Four stop codons were found in the mt genomes of the two *Dactylis* species. These included TAA, which was detected in 11 genes (*atp6*, *atp8*, *cox2*, *nad1*, *nad2*, *nad3*, *nad4L*, *nad5*, *nad6*, *nad9*, *rps4*, and *rps7*), TGA found in eight genes (*atp1*, *atp9*, *ccmB*, *cox3*, *nad4*, *rps1*, *rps12*, and *rps13*), TAG detected in nine genes (*atp4*, *ccmC*, *ccmFn*, *cob*, *cox1*, *matR*, *mttB*, *nad7*, and *rps3*), and CGA detected in one gene (*ccmFc*). Leucine (Leu), serine (Ser), and isoleucine (Ile) were the most abundant amino acids, while cysteine (Cys) was the least abundant amino acid in the two species, similar to most angiosperms (Fig. 2).

We further analyzed the relative values of synonymous codon usage (RSCU) and found that the values increased with the number of codons (Fig. S5). Almost all the amino acid codons had a bias (RSCU > 1 or RSCU < 1) in two *Dactylis* mt genomes, except for tryptophan (UGG, RSCU = 1). Most preferred codons (RSCU > 1) ended with A or U, except the UUG codon. This phenomenon may be due to the preference of the A/U-ending codons by monocots, indicating the potential role of natural selection and mutation in the evolution of *Dactylis*.

### Analysis of the RNA editing sites in the PCGs

**Fig. 3:**
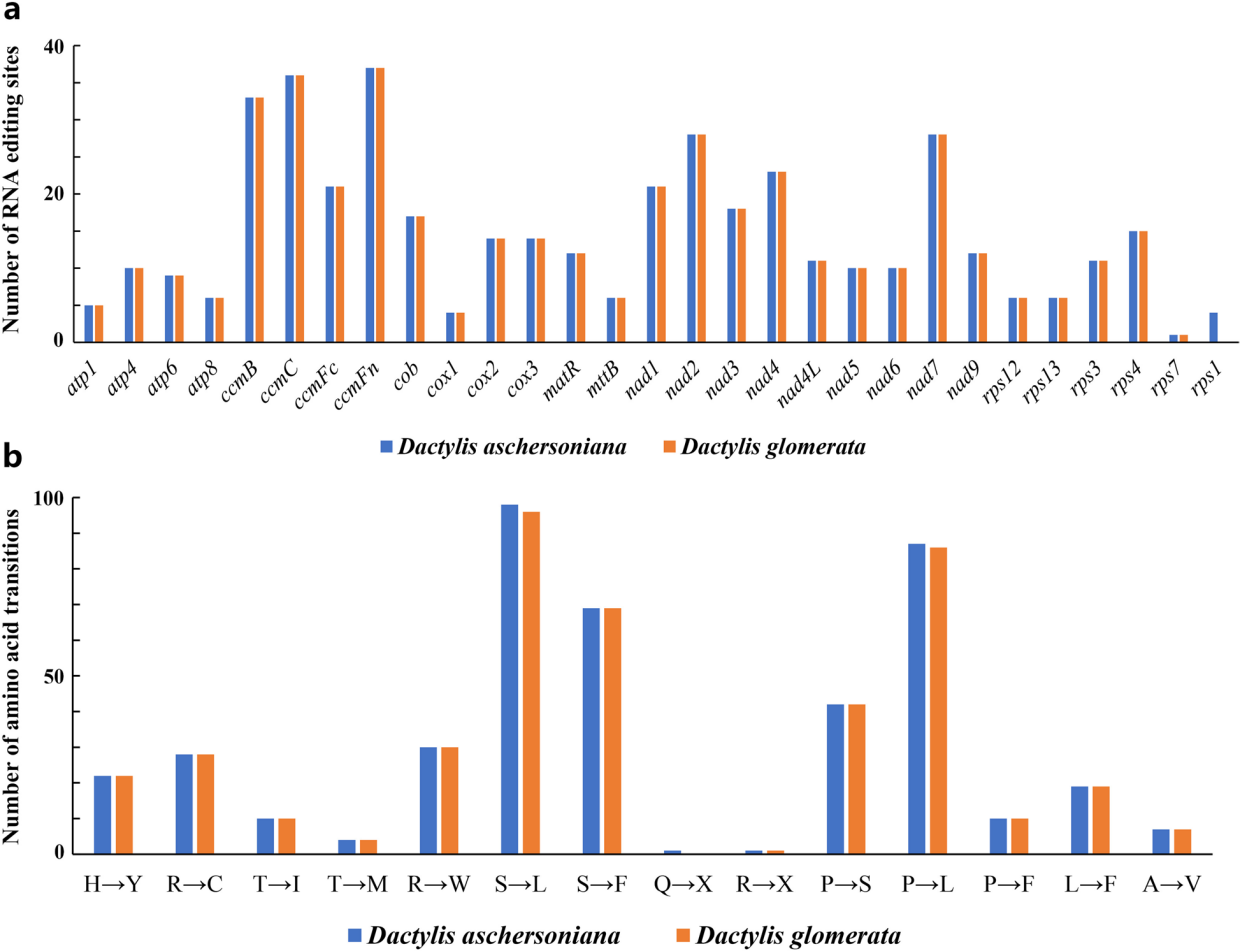
RNA editing in the mitochondrial (mt) genomes of *Dactylis aschersoniana* and *Dactylis glomerata*. **a** The number of RNA editing sites. **b** The number of amino acid transitions

RNA editing is one of the post-transcriptional modifications necessary to maintain gene expression in the cp and mt genomes of higher plants. Previous studies showed that converting cytosine to uridine after RNA editing can alter genomic information [26]. To provide a theoretical basis for studying RNA editing of mt genes in *Dactylis*, we predicted the potential RNA editing sites in the mt genomes of *D. glomerata* and *D. aschersoniana*. The RNA editing analyses revealed the presence of 424 RNA editing sites in 28 genes and 428 RNA editing sites in 29 genes in the mt genomes of *D. glomerata* and *D. aschersoniana*, respectively (Fig. 3a). Further analysis of the RNA editing sites revealed that these RNA editing events were edited on the first and second bases of the codons, with the frequency of second base editing being much higher. Notably, 424 editing sites were common to the two *Dactylis* species, whereas four editing sites in *rps1* were specific to *D. aschersoniana*. Moreover, the number of RNA editing sites was unbalanced between genes. Cytochrome c biogenesis genes (*ccmB*, *ccmC*, and *ccmFn*) and NADH dehydrogenase genes (*nad1*, *nad2*, *nad4*, and *nad7*) had the highest number of RNA editing sites in the mt genomes of the two *Dactylis* species. In contrast, the genes encoding transport membrane protein (*mttB*), ATP synthase (*atp1*, *atp4*, *atp6*, and *atp8*), and ribosomal proteins (SSU) (*rps7*, *rps12*, *rps13*, and *rps1*) exhibited the lowest number of RNA editing sites. RNA editing can cause changes in the start codons and stop codons of the PCGs. As shown in Table S5, *nad1* and *nad4L* genes had ACG as the start codon, which was changed to AUG after RNA editing. In addition, *ccmFc* had CGA as the stop codon, which was modified to UGA after RNA editing.

Moreover, our results showed that the codons of amino acids tend to encode leucine after RNA editing. In particular, serine-to-leucine conversion was the most frequent, followed by proline-to-leucine (Fig. 3b). This study also found that the hydrophilicity and hydrophobicity of amino acids could change after RNA editing (Table S5). The hydrophobicity of 49.29%-49.30% of the amino acids changed from hydrophilic to hydrophobic, while that of 9.81%-9.91% changed from hydrophobic to hydrophilic. However, the hydrophobicity of 28.74%-28.77% of the amino acids did not change. This indicated that RNA editing significantly increased the hydrophobicity of the mt proteins.

### Analysis of the repeat sequences

Repeat sequences are an important source for developing population and evolutionary analysis markers. SSRs, tandem repeats and long repeats are widely distributed in plant mt genomes. Repeat-mediated homologous recombination can generate structural variation and extreme mt genome sizes. Thus, SSRs, tandem repeats, and dispersed repeats were analyzed in this study. There were differences in the number of SSRs in the mt genomes of the two *Dactylis* species, which ranged from 558 (*D. glomerata*) to 564 (*D. aschersoniana*) (Table S6). Six types of SSRs were detected in the two *Dactylis* species, including monomer, dimer, trimer, tetramer, pentamer, and hexamer repeats (Table S6), and the number of these SSR types varied widely. The most abundant SSRs were trimer repeats (51.43%-52.48%), followed by monomer repeats (29.61%-31.18%) and tetramer repeats (8.60%-8.87%). Pentamer and hexamer repeats were very rare in the two mt genomes and accounted for 3.05%-3.19% and 0.89%-1.25% of the SSR repeats, respectively (Table S6). Nearly all monomer repeats (25.35%-26.34%) were composed of A and T bases in these two *Dactylis* species, and the trimer repeats of AAG/CTT were the second most common SSRs (20.97%-21.53%) (Table S7). A total of 14 (*D. glomerata*) and 21 (*D. aschersoniana*) tandem repeats with lengths ranging from 5 to 43 bp and 100% of sequence identity were identified in the two mt genomes (Table S8). The most abundant types of tandem repeats identified in *D. aschersoniana* and *D. glomerata* mt genomes were the small tandem repeats, with a length of 5–43 bp. However, a long tandem repeat sequence (81 bp) was also detected in the *D. glomerata* mt genome. In addition, we detected dispersed repeat sequences with 100% identity. A total of 50 dispersed repeat sequences were detected in the *D. aschersoniana* mt genome, including 22 forward repeats and 28 palindromic repeats (Table S9). Conversely, the mt genome of *D. glomerata* contained 60 dispersed repeat sequences, including 30 forward repeats and 30 palindromic repeats. Long repetitive sequences were also detected in the mt genomes of *D. aschersoniana* and *D. glomerata*. In the mt genome of *D. glomerata*, four repeats (R1, R10, R11, and R12) were larger than 1 kb, with the largest repeat being R10 (7180 bp). However, only two repeats larger than 1 kb (R11: 5991 bp and R1: 3726 bp) were detected in the mt genome of *D. aschersoniana*. The mt genome of *D. aschersoniana* contained more short repetitive sequences than that of of *D. glomerata*, indicating that short repetitive sequences may expand its mt genome.

### DNA transfer from chloroplast to mitochondria

**Fig. 4:**
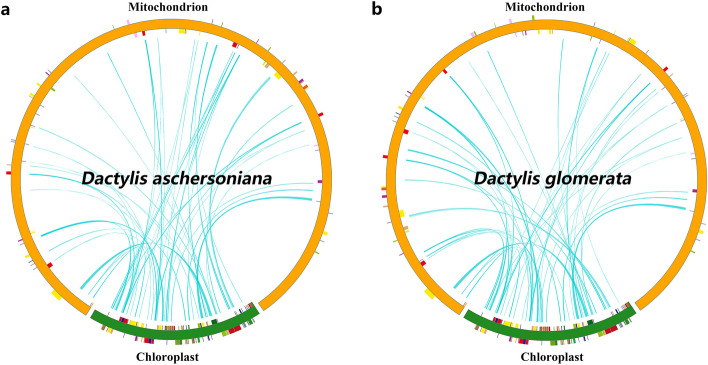
Schematic representation of the chloroplast-to-mitochondrial gene transfer. The chloroplast-to-mitochondrial gene transfer in **a** *Dactylis aschersoniana* and **b** *Dactylis glomerata*

The length of the mt genomes of *D. aschersoniana* and *D. glomerata* (597, 281–613, 769 bp) was approximately 4.5 times longer than that of their corresponding cp genomes (134, 972–134, 986 bp) (Fig. 1). In the mt genome of *D. aschersoniana*, 53 fragments with a total length of 23, 543 bp, accounting for 3.8% of the mt genome, had relocated from the cp genome to the mt genome. Similarly, 52 fragments with a total length of 23, 860 bp, accounting for 4% of the mt genome, had relocated from the cp genome to the mt genome of the *D. glomerata* (Fig. 4 and Table S10). The sequence identity of these fragments was more than 70%. Furthermore, two intact cp genes (*petN* and *psbM*) and nine tRNAs (*trnL-CAA*, *trnF-GAA*, *trnH-GUG*, *trnP-UGG*, *trnN-GUU*, *trnC-GCA*, *trnW-CCA*, *trnM-CAU*, and *trnS-GCU*) were shared between the cp and mt genomes, and partial sequences of the remaining four genes (*ndhJ*, *ndhK*, *rpl14*, and t*rnV-GAC*) were also identified. Interestingly, two tRNAs (*trnS-GGA* and *trnH-GUG*) were localized on the mt genome fragments of *D. glomerata,* and only one tRNA, *trnS-GCU*, was localized on the mt genome fragments of *D. aschersoniana* (Table S10). Notably, the migration of the cp genes was heterogeneous, with the large-single copy (LSC) region being higher than the inverted repeat (IR) region.

### Phylogenetic and selective pressure analysis

**Fig. 5:**
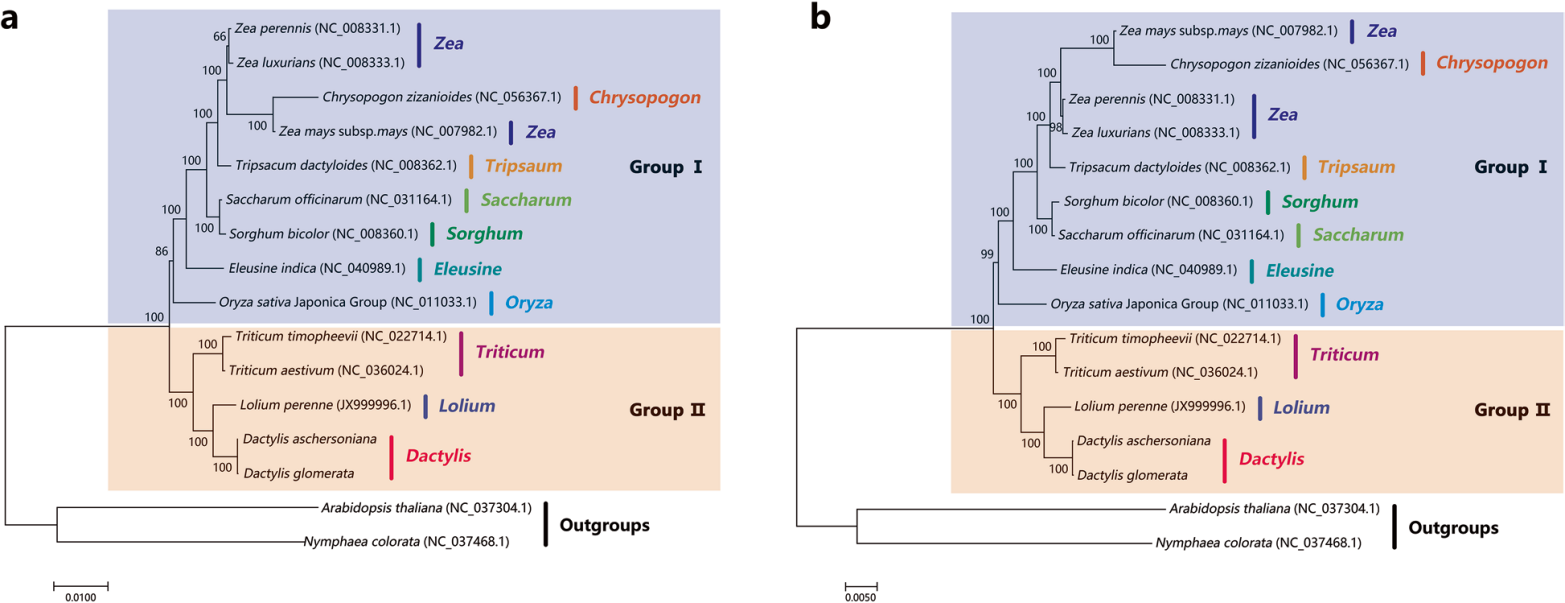
Phylogenetic analysis of the Gramineae species. **a** Phylogenetic tree constructed using the maximum-likelihood method. **b** Phylogenetic tree constructed using the Bayesian method

To better understand the phylogenetic relationship between the *Dactylis* species and other Gramineae plants, we compared the mt genomes of the two *Dactylis* species with that of the 12 other Gramineae plants (Fig. 5). The phylogenetic tree was constructed using 29 protein-coding sequences, including *atp1*, *atp4*, *atp8*, *atp9*, *ccmB*, *ccmC*, *ccmFn*, *ccmFc*, *cob*, *cox1*, *cox2*, *cox3*, *matR*, *mttB*, *nad1*, *nad2*, *nad3*, *nad4*, *nad4L*, *nad5*, *nad6*, *nad7*, *nad9*, *rps1*, *rps12*, *rps13*, *rps3*, *rps4*, and *rps7*. *Arabidopsis thaliana* and *Nymphaea colorata* were used as the outgroups for the phylogenetic analysis. The sequences clustered into two groups. Group I had a high bootstrap support (BS). In group II, five Pooideae species (*D. aschersoniana*, *D. glomerata*, *Lolium perenne*, *Triticum aestivum*, and *T. timopheevii*) were clustered in one clade with high bootstrap values (100%). The phylogenetic tree showed that the genus *Dactylis* was closely related to *Lolium perenne*. Furthermore, the phylogenetic analysis revealed that the genus *Dactylis* formed a close genetic relationship with two species of the genus *Triticum*, consistent with the previous phylogenetic tree based on the cp genome, thus indicating that the results of the mt genome were reliable [25].

**Fig. 6:**
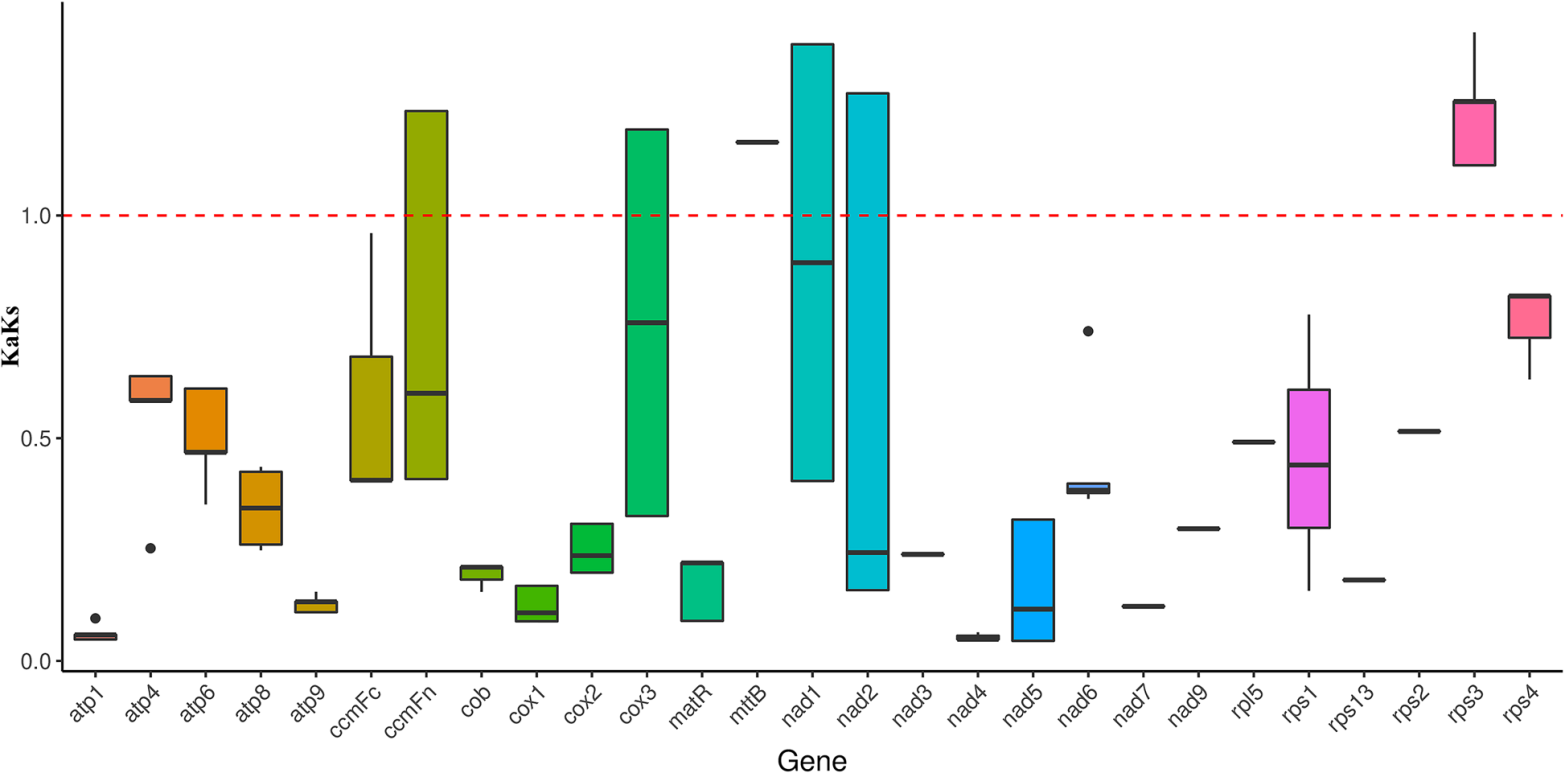
The non-synonymous/ synonymous (Ka/Ks) ratio analysis of 27 coding genes common among *Dactylis aschersoniana*, *Dactylis glomerata*, and two other species

Calculating the Ka/Ks values is crucial for reconstructing phylogenetic trees and studying the evolutionary patterns of PCGs among closely related species (Fig. 6). In general, Ka/Ks ratios > 1.0, = 1.0, < 1.0 represent positive, neutral, and stable selections, respectively. Importantly, the Ka/Ks ratio cannot be significantly higher than 1.0 without at least some favorable mutations. Here, we calculated the Ka/Ks values of 27 PCGs in the mt genomes of four Pooideae species, namely, *D. aschersoniana*, *D. glomerata*, *L. perenne*, and *T. aestivum*. The Ka/Ks ratio was very low (approaching zero) between the shared PCGs in the mt genomes of *D. aschersoniana* and *D. glomerata*. In contrast, the Ka/Ks ratios of 21 out of the 27 PCGs shared by the mt genomes of the four Pooideae species were < 1.0, indicating that these PCGs were stably selected during evolution. Thus, several mt genes that had undergone stable selection may play an important role in stabilizing the normal mitochondrial function. Six genes (*ccmFn*, *cox3*, *mttB*, *nad1*, *nad2*, and *rps3*) had Ka/Ks > 1.0, indicating they had undergone positive selection after differentiating from their last common ancestor. The results showed that *rps3* had the highest ratio, followed by *nad1*, *nad2*, and *ccmFn*. The high Ka/Ks ratio of these genes may be important for the evolution of Pooideae species.

### Homology analysis and genome rearrangement events

**Fig. 7:**
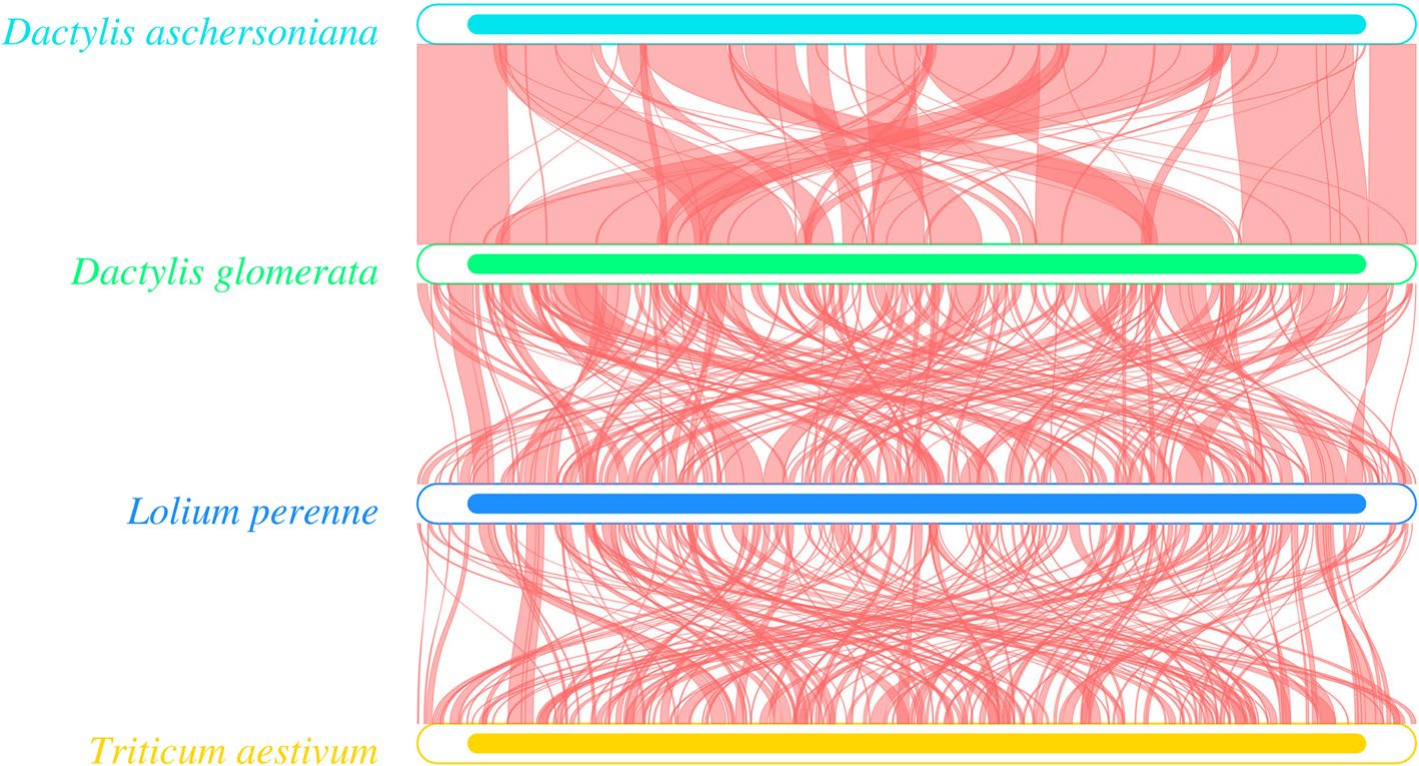
Collinearity analysis of the mitochondrial genomes. Collinearity analysis of the mitochondrial genomes of *Dactylis aschersoniana*, *Dactylis glomerata* and two other Gramineous species

Homology analysis of four Pooideae plants showed no diagonal oblique line in the lattice diagram of *D. aschersoniana* and *D. glomerata*, but several oblique lines parallel to the diagonal. The oblique line showed that the two sequences had the same substring, indicating that the mt genome sequences of *D. aschersoniana* and *D. glomerata* were slightly different with lower homology (Fig. S6). In addition, the mt genome sequences of *L. perenne*, *T. aestivum*, and the two *Dactylis* species were more different, indicating that the mt genome varied significantly in Gramineous plants. Thus, the results of the mt genome homology analysis supported the theory that the mt genome varies greatly within the same plant species. The arrangement of the mt genes has been widely used to understand the phylogenetic status between species. To evaluate the mt genome rearrangement, we compared the mt genomes of four Gramineous species, namely *D. aschersoniana*, *D. glomerata*, *L. perenne*, and *T. aestivum*. Several local collinear blocks were observed in the mt genomes of *D. aschersoniana*, *D. glomerata*, *L. perenne*, and *T. aestivum* (Fig. 7). The size and position of these local collinear blocks varied greatly among the four Gramineous species. Rearrangement analysis showed that several rearrangements had occurred between the mt genomes of the two *Dactylis* species and *T. aestivum*. Notable, there were even several gene rearrangements between the mt genomes of the two *Dactylis* species, indicating that the mt genomes of Pooideae plants are highly different. We also found some consistency between the results of gene rearrangement and phylogenetic analysis. Species that shared more homologous sequences tended to have closer relationships in phylogenetic trees, such as the mt genomes of *D. aschersoniana, D. glomerata*, and *L. perenne*.

## Discussion

Since the first endosymbiotic events, the size and structure of plant mt genomes have undergone rapid and dramatic changes [27, 28], making the composition of the plant mt genomes extremely complex. These genome changes have rendered the traditional sequencing and assembly methods inefficient, making the study of plant mt genomes challenging [29, 30]. However, the tremendous advancement of sequencing technologies in the past years has greatly promoted the study of plant mt genomes. This study presents a novel strategy for obtaining the plant mt genome, which combines the second-and third-generation whole-genome sequencing data and leverages the higher copy number of plant organelle genome compared to the corresponding nuclear genome [24]. Thus, this study sequenced, annotated, and reported the complete mt genomes of *Dactylis* species for the first time. Combining Illumina and PacBio sequencing technologies overcomes the assembly problem of such complex genomes, thus providing a reference for future mt genome research. Compared with animal mt genomes, plant mt genomes exhibit multiple structures, including circular, linear, branched, and mixed forms [27]. Based on previous research, most mt genomes are circular, and only a few mt are linear [31]. The assembled sequences of the two *Dactylis* mt genomes were typical circular DNA molecules, with genome sizes ranging from 587, 289 bp (*D. glomerata*) to 613, 769 bp (*D. aschersoniana*), indicating that the genome size could be very different even among species of the same genus. According to previous reports, *Dactylis* species have the largest mt genome among the Pooideae plants published on NCBI, including *Elymus sibiricus* (347, 265 bp), *T. aestivum* (452, 526 bp), and *T. timopheevii* (443, 419 bp) [32].

Similar to most plants, the non-coding sequences in the intergenic region of the mt genomes of *D. glomerata* and *D. aschersoniana* represented a substantial proportion of the mt genome, accounting for 88.79%-89.10% of the total mt genome [33]. This indicated that non-coding sequences may be the main source of mt genome variations [34]. In general, non-coding sequences may consist of many repeat sequences, including transition sequences of the cp and nuclear genome and the sequences horizontally transferred from other species [35]. Repeat sequences include short repeats, tandem repeats, and long repeats. Repeat-mediated homologous recombination is almost ubiquitous in plant mt genomes, and this phenomenon greatly increases the size of the mt genome [10]. In this study, *D. aschersoniana* had a larger mt genome and contained many short repetitive sequences than *D. glomerata*, indicating that the size of the *D. glomerata* mt genome is related to the accumulation of the short repetitive sequences. Short repeat sequences are also very important for the structural evolution of mt genomes in higher plants since their accumulation led to the expansion of mt genomes in plants such as *Zucchini* [36]. Our study inferred that the size of the mt genome is related to the short repeat sequences.

One of the key events determining the size of the mt genome in angiosperms is the frequent sequence transfer from the cp genome to the mt genome, accounting for 1–12% of the total genomic length [22]. In general, the frequency of DNA transfer events from the mt genome to the cp genome is low because the cp genome is considered to be highly conserved and lacks effective DNA uptake mechanisms [37]. Although the transfer of DNA sequences from cp to mt genomes is a common occurrence, the size of transferred DNA varies among higher plant species, ranging from 50 kb in *Arabidopsis* to 1.1 Mb in *Oryza sativa subsp.japonica* [38]. Recent studies revealed that frequent DNA transfer from cp to mt genomes of the common ancestor of gymnosperms and angiosperms occurred as early as approximately 300 million years ago. Furthermore, the transfer of DNA sequences from cp was positively correlated with the size of mt genomes [39]. The presented study found that the total length of DNA sequences transferred from cp genomes to mt genomes accounted for 3.8%-4% of the total mt genome. Furthermore, the amount of DNA in Legume *Vigna* (0.5%) and *Acer truncatum* (2.36%) was much lower than *D. aschersoniana* and *D. glomerata* [14, 40], indicating that the transfer rates of the cp sequences may be common in *D. aschersoniana* and *D. glomerata*. Meanwhile, our results revealed that the total length of cp-derived sequences in the *D. glomerata* mt genome (a smaller mt genome) was 23, 860 bp, while the total length of cp-derived sequences in the *D. aschersoniana* mt genome (a larger mt genome) was 23, 543 bp. This indicated that there is no relationship between the size of the mt genome and the amount of cp-derived sequences. This phenomenon was also observed in *Cucurbitaceae* plants [40]. Approximately 79 kb of the cp migration sequences were integrated into the melon mt genome (the largest mt genome), and about 113 kb of the cp migration fragments were integrated into the *Zucchini* mt genome, which is smaller than that of melon [35]. The study found that half of the sequences of the melon mt genes were similar to that of the nuclear genome. Moreover, it was found that the amplification of the mt genome of *Cucurbitaceae* plants is related to the migration sequences of the nuclear genome. Thus, it can be tentatively speculated that the expansion of the mt genome of *Dactylis* may be related to the accumulation of short repeat sequences and nuclear genome migration sequences. However, the specific cause remains to be further investigated by assembling the nuclear genome of *D. aschersoniana* and *D. glomerata*.

An additional source of sequence variation in the mt genomes of land-plant lineages may be attributed to RNA editing, which occurs during the post-transcriptional modifications of higher plant genomes [26]. The first instance of editing in land plant plastome was identified in the *rpl2* gene of the cp genome of maize in 1991 [41]. Since then, numerous RNA editing sites have been discovered in various plants, including *Arabidopsis*, which contains 36 RNA editing sites in 441 genes [42], and *O. sativa*, which has approximately 491 RNA editing sites in 34 genes [43]. In general, RNA editing usually occurs in the first and second bases of codons, facilitating the creation of appropriate secondary protein structures by generating novel start and stop codons or altering amino acid sequences. This process is crucial for gene regulation and the precise expression of genetic information within cells [44]. In this study, *nad1* and *nad4L* genes had ACG as the start codon, which was changed to AUG after RNA editing. This indicated that RNA editing generated the AUG start codons of the *nad1* and *nad4L* mRNAs required for protein synthesis. Moreover, the generation of *nad1* and *nad4L* start codons may indicate the regulatory role of the editing process in achieving the conversion of non-functional mRNA into translatable mRNA. The AUG start codon has been shown to be generated by editing the ACG codons in maize cp *rpl2* and wheat mt *nadl* mRNA [45]. In addition, several studies suggested that many important cultivation traits are closely related to mt RNA editing, such as the ripening mechanism of tomato fruits and the length of cotton fiber [46, 47]. Compared with *O. sativa* and *Arabidopsis*, the presented study detected 424 RNA editing sites in 28 and 428 RNA editing sites in 29 genes (*rps1*) in the mt genomes of *D. glomerata* and *D. aschersoniana*, respectively. The reduced number of RNA editing sites observed in the mt genomes of the genus *Dactylis* might have been due to the reduction in the number of mt genes. Furthermore, a previous study reported the relationship between *rps1* and heat tolerance in plants [17]. Previous studies found that the cp ribosomal protein, *rps1*, is an essential factor regulating the retrograde activation of the heat stress response in higher plants, thus possibly acting as a coordinator of retrograde communication to trigger nuclear gene expression that is crucial for heat tolerance [17]. However, *rps1* responds to heat stress at the protein level but not at the transcriptional level and, thus, might not be identified as a heat-responsive protein via heat-responsive transcriptome analysis. Our study found that RNA editing of the *rps1* gene occurred only in the mt genome of *D. aschersoniana*. Therefore, we speculated that the *rps1* gene in the mt genome of *D. aschersoniana* might have been transformed from non-functional mRNA into translatable mRNA via RNA editing, thereby improving the heat resistance of *D. aschersoniana*. Thus, identifying these RNA editing sites could provide crucial information for predicting the function of genes containing new codons, which can help to better understand the gene expression of the plant mt genome.

Since the 1990s, there have been tremendous advances in molecular biology techniques. The utilization of PCR, traditional Sanger sequencing, and NGS technology has significantly advanced various biological fields, including phylogenetic research. The phylogenetic framework and the categorization of the specific lineages of angiosperms were initially based on gene construction from the cp (*atpB*, *matK*, *rbcL*) and nuclear (18S rDNA) genomes [48–50]. However, recent studies also explored the third class of DNA-containing organelle, the mitochondria, establishing the potential of mt genes to solve phylogenetic problems at different plant classification levels [51, 52]. In this study, 29 PCG sequences of the mt genome were used to illustrate the preliminary relationships among the selected representatives of gramineous plants. The clustering results of the phylogenetic tree based on the mt genomes of *Dactylis* species and that of the 12 other Gramineae plants were surprisingly consistent with the previous studies based on cp genome sequences. This demonstrated the possibility of using information obtained from the mt genome in plant phylogenetic studies. The phylogenetic tree constructed in this study revealed that *Dactylis* species were more closely related to *L. perenne* than to *T. aestivum*. This observation, in conjunction with the similarity with the phylogenetic tree derived from cp genomic data, demonstrated the reliability of the results and confirmed the significance of mt genes in phylogenetics [25]. In addition to the genus, family, or other higher taxa of angiosperms, these underutilized DNA markers can also be used to evaluate the relationships at the interspecific level.

To evaluate the selection pressure in the evolutionary dynamics of the PCGs among closely related species, we utilized the Ka/Ks ratio, a crucial metric for investigating the evolutionary dynamics of PCGs in related species [53, 54]. Ka/Ks analysis of the mt genomes of *D. aschersoniana*, *D. glomerata*, *L. perenne*, and *T. aestivum*, showed that the PCGs of *D. aschersoniana* and *D. glomerata* were conserved. These results indicated that mt genes were highly conserved during the evolution of land plants. However, some PCGs, including *ccmFn*, *cox3*, *mttB*, *nad1*, *nad2*, and *rps3*, exhibited a Ka/Ks value greater than 1, indicating positive selection during their evolution. These findings underscore the significance of genes with high Ka/Ks ratios in the selection and evolution of angiosperm genes.

The arrangement of mt genes has been widely used to understand the phylogenetic relationship between species. Since the mt genome of some species in *Dactylis* has not been reported, this study only compared the collinearity of the complete mt genomes of two *Dactylis* species and two other Pooideae species to evaluate the degree of structural rearrangement between different species. Gene order comparison often reflects the rate of mt genome rearrangement among plant species. We found that mt gene rearrangements occurred widely in these four species, consistent with many previous studies on plant mt genomes [55, 56]. Homology analysis showed low sequence similarity with a short period of homology when compared between species. These rearrangement events suggest that the gene order is more conserved in closely related than in more distantly related species. In general, species with close evolutionary relationships share more homologous blocks [36, 55]. For example, higher sequence similarity was found between *D. aschersoniana*, *D. glomerata*, and *L. perenne* than between *D. aschersoniana*, *D. glomerata*, and *T. aestivum*. Thus, our results lay the foundation for further analysis of the evolutionary relationships of gramineous plants. However, due to the lack of sufficient representative mt genomes, more mt genomes need to be sequenced to better understand the phylogeny and evolution of gramineous plants.

## Conclusions

This study assembled and annotated mt genomes of the genus *Dactylis* for the first time using the PacBio sequencing technology. The mt genomes of *D. aschersoniana* and *D. glomerata* showed a typical circular structure with a genome size of 597, 289 bp and 613, 769 bp, respectively. The large genomic sizes may be due to the accumulation of many short repeat sequences. Codon bias, RNA editing, and gene transfer between cp and mt were also analyzed. Ka/Ks analysis showed that most mt genes underwent stable selection, indicating that most mt genes were conserved during evolution. The phylogenetic tree constructed using conserved PCGs showed that the evolution of the mt genome was consistent with that previously reported based on the cp genome. In addition, homology analysis showed that species with close evolutionary relationships shared more homologous blocks. These results will facilitate further characterization of the mt genome of *Dactylis* and provide a reference for determining the evolutionary relationships of Gramineae plants.

## Methods

### Plant material and DNA extraction

Two *Dactylis* species, AKZ-NRGR667 and D20170203, were used in this study. The seeds of AKZ-NRGR667 (Registered No. AKZ-NRGR667) were obtained from the National Plant Germplasm System (NPGS), USA, while those of D20170203 (No. D20170203) were obtained from the Department of Grassland Science, Sichuan Agricultural University, China. The plants were asexually propagated through tiller buds and grown in the greenhouse of Sichuan Agricultural University (30°42'N, 103°51'E) Chengdu, Sichuan Province, China. The lighting and temperature conditions were 14 h/10 h (day/night) and 22 °C/15 °C (day/night), respectively (Table S11). Fresh leaves were collected at the three-leaf stage and stored at -80 °C. A TIANGEN Plant Genomic DNA Kit (DP305) was used to obtain high-quality genomic DNA.

### Chloroplast genome sequencing, assembly and annotation

The cp genomes of the two *Dactylis* species were sequenced and assembled using the reference sequencing and assemblage strategy [25], with a sequencing read length of PE150. After sequencing, Fastp (v0.20.0, https://github.htm) was employed to remove adapters and low-quality sequences [57]. The coding sequences (CDS), ribosomal RNA (rRNA), and transfer RNA (tRNA) were then annotated using prodigal v2.6.3 [58], hmmer v3.1b2 [59], and ARAGORN v1.2.38 [60]. In addition, BLAST v2.6 was utilized to extract cp genomic data from the NCBI database for alignment with the assembled sequences [61]. Finally, manual correction was performed to eliminate incorrect and redundant annotations and intron/exon boundaries. Circular maps of all the cp genomes were drawn using the program OGDRAW v1.1 [62].

### Mitochondrial genome sequencing, assembly, and annotation

The third-generation sequencing data were assembled using the third-generation assembly software Canu to obtain the contig sequence. We used the contig sequence to search the plant mt gene database using the BLAST v2.6 [61]. The aligned mt gene contig was used as the seed sequence, and the original data was used for extension and cyclization to finally reveal its ring structure. The final assembly result was obtained by manually correcting the errors of the second and third-generation assembly data using NextPolish1.3.1 [63]. Sequence matches were identified via BLAST searches and compared with previously reported plant mt genomic sequences. Closely related species were manually modified based on encoded proteins and rRNAs. The tRNAs were annotated using the tRNAscanSE program [64], while the Open Reading Frame Finder was used to annotate the open reading frames (ORFs) [65]. The mt genome was assembled using the OrganellarGenomeDRAW program [62].

### The generation of sequencing depth and coverage map for organelle genome

The sequencing depth and coverage map are crucial for the sequencing and analysis of organelle genomes. To assess the integrity and accuracy of chloroplast and mitochondrial genomes, we conducted the sequencing depth and coverage map generation for organelle genomes [66].

### Comparative genome analysis

The mitochondrial genomes from *Dactylis aschersoniana* and *Dactylis glomerata* were aligned in mVISTA with *Dactylis aschersoniana* as a reference [67].

### Repeat element analysis

Repeat sequences were classified into three: SSRs, tandem repeats, and dispersed repeats. SSRs were identified using MISA software (v1.0, parameters: 1–10, 2–5, 3–4, 4–3, 5–3 and 6–3) [68], while tandem repeats were identified using Tandem Repeats Finder software (trf409.linux64, parameters: 27, 7, 80, 10, 50, 2000-f-d-m) [69], and dispersed repeats were identified using BLASTN (v2.10.1, parameters: -word size 7, e-value 1e-5, remove redundancy, remove tandem repeats) [70]. The repeats were visualized with Circos software v0.69–5 [71].

### Condon preference analysis

Codon preference is considered a comprehensive result of natural selection, species mutation and genetic drift. It is calculated by the method: (the number of one codon encoding an amino acid/the number of all codons encoding the amino acid)/ (1/the type of codon encoding the amino acid)/ (the actual usage frequency of the codon/the theoretical usage frequency of the codon). We used our own Perl script to filter and calculate the CDS.

### Identification of chloroplast gene insertion in the mitochondria

DNA migration is common in plants and occurs during autophagy, gametogenesis and fertilization. The BLAST tool was used to find the homologous sequences between cp and mt of orchardgrass in the NCBI database. The similarity was set to ≥ 70%, the E-value was ≤ 1e-5, and the length was ≥ 40. The obtained sequences were visualized with Circos v0.69–5 [69].

### Prediction of the mitochondrial RNA editing sites in orchardgrass

The plant predictive RNA editor (PREP) was used to determine the RNA editing sites in the *Dactylis* mt genome, with the critical value set to 0.2 [72].

### Ka (non-synonymous)/Ks (synonymous) ratio analysis

Mafft v7.310 was used for gene sequence alignment [73], and the values of Ka, Ks, and Ka/Ks were estimated using the KaKs Calculator v2.0 [74], with MLWL as the calculation method. The ratio of non-synonymous mutation rate (Ka) to synonymous mutation rate (Ks) greater than 1 indicates a positive selection effect and less than 1 indicates a purified selection effect.

### Collinearity analysis

In this study, two methods were used for collinearity analysis. The first method utilized nucmer (MUMmer4, 4.0.0beta2) and-maxmatch parameter to perform genome alignment between the sequences of other Pooideae species and the assembled orchardgrass mt genome, after which a dot plot diagram was generated [75]. The second method used BLASTN [70], with the E-value set to 1e-5, for screening the fragments with a length greater than 300 bp. The assembled orchardgrass genomes and the selected Pooideae species were compared to generate a collinearity map.

### Phylogenetic analysis

The 29 CDSs common among the species were used to construct phylogenetic tree. Sequences were compared between the species using MAFFT software (v7.427, -auto mode) [73]. The sequences with good alignment were joined at the beginning and end and were trimmed using trimAl (v1.4.rev15) (parameter: -gt 0.7) [76]. After trimming, jModelTest-2.1.10 software was used to predict the model, which was determined to be of the GTR type [77]. Thereafter, the GTRGAMMA model of the RAxML v8.2.10 was used to construct the maximum likelihood phylogenetic tree with 1000 bootstrap replicates [78]. Bayesian inference (BI): Each set of CDS sequences underwent multiple sequence alignment using MAFFT v7.427 software (–auto mode). The concatenated sequences were then analyzed using MrBayes v3.2.7a software [79]. The GTR + I + G model was seletcted, with Ngammacat set to 5. Statefreqpr, revmat, pinvar, and shapepr were set based on the best model identified by the jModelTest software, while the remaining parameters were kept at default settings. The concatenated sequences were then analyzed using MrBayes v3.2.7a software.

### Supplementary Information

**Additional file 1: Fig. S1.** The information of sequencing data. a and b, The raw data of the third-generation sequencing read length distribution of *Dactylis aschersoniana* and *Dactylis glomerata*, respectively; c and d, The A/T/G/C content distribution statistics of *Dactylis glomerata *and* Dactylis aschersoniana*, respectively. **Fig. S2.** Base error rate and quality distribution. a and b, The error rate distribution of *Dactylis aschersoniana* and *Dactylis glomerata*, respectively; c and d are quality distribution of *Dactylis aschersoniana* and *Dactylis glomerata*, respectively. **Fig. S3.** Sequencing depth and coverage map of chloroplast and mitochondrial genomes. a and b represent the sequencing depth of coverage map from the chloroplast genomes of *Dactylis aschersoniana* and *Dactylis glomerata*. c and d represent the sequencing depth of coverage map from the mitochondrial genomes of *Dactylis aschersoniana* and *Dactylis glomerata*. **Fig. S4.** The sequence identity maps of two *Dactylis* mitochondrial genomes. The gray arrow above the alignment indicates the direction of the gene. Blue stripes represent exons, and pink stripes represent non-coding sequences (CNSs). The graph uses a critical value of 50 % identity. The Y axis represents the identity percentage in the range of 50-100 %. **Fig. S5.** Codon distribution map in the Dactylis mt genome. Red indicates a high relative synonymous codon usage (RSCU) value and green indicates a low RSCU value. Hierarchical clustering (average linkage method) was performed for the codon patterns (x-axis). **Fig. S6.** The base sequence dot-plot diagram of *Dactylis aschersoniana*, *Dactylis glomerata* and other two species.

**Additional file 2: Supplementary Table S1.** Second generation sequencing data. **Supplementary Table S2.** Third generation sequencing data. **Supplementary Table S3.** Genome structure within mitochondrial genomes of two *Dactylis* species. **Supplementary Table S4.** Gene organization of the mitochondrial genomes of two *Dactylis *species. **Supplementary Table S5.** Prediction of RNA editing sites in *Dactylis* species. **Supplementary Table S6.** Frequency of classified SSR types in the mitochondrial genomes of two *Dactylis *species. **Supplementary Table S7.** Frequency of classified repeat types in *Dactylis glomerata*. **Supplementary Table S8.** The distrubution of tandem repeat sequence in the mt genome of Dactylis. **Supplementary Table S9.** Distribution of interspersed repeats in two mt genome. **Supplementary Table S10.** Fragments transferred from chloroplasts to mitochondria. **Supplementary Table S11.** The material information of *Dactylis*.

## Data Availability

The raw data supporting the conclusions of this article have been de–posited into the CNGB Sequence Archive (CNSA) of the China National Gene Bank Data Base (CNGBdb) with accession number CNP0003657, (https://db.cngb.org/search/?q=CNP0003657). The plant materials were provided by the Department of Forage Science, College of Grassland Science and Technology, Sichuan Agricultural University, Chengdu, China.

## Abbreviations

mt: Mitochondrial
PCGs: Protein-coding genes
tRNAs: Transfer RNAs
rRNAs: Ribosomal RNAs
cp: Chloroplast
SSRs: Simple sequence repeats
