# Supplementary material for: The first two whole mitochondrial genomes for the genus Dactylis species: assembly and comparative genomics analysis
Source: BMC Genomics. 2024 Mar 4;25:235. doi: 10.1186/s12864-024-10145-0 (PMC10910808; doi:10.1186/s12864-024-10145-0)
Supplement: Supplementary file 1 — Additional file 1: Fig. S1. The information of sequencing data. a and b, The raw data of the third-generation sequencing read length distribution of Dactylis aschersoniana and Dactylis glomerata, respectively; c and d, The A/T/G/C content distribution statistics of Dactylis glomerata and Dactylis aschersoniana, respectively. Fig. S2. Base error rate and quality distribution. a and b, The error rate distribution of Dactylis aschersoniana and Dactylis glomerata, respectively; c and d are quality distribution of Dactylis aschersoniana and Dactylis glomerata, respectively. Fig. S3. Sequencing depth and coverage map of chloroplast and mitochondrial genomes. a and b represent the sequencing depth of coverage map from the chloroplast genomes of Dactylis aschersoniana and Dactylis glomerata. c and d represent the sequencing depth of coverage map from the mitochondrial genomes of Dactylis aschersoniana and Dactylis glomerata. Fig. S4. The sequence identity maps of two Dactylis mitochondrial genomes. The gray arrow above the alignment indicates the direction of the gene. Blue stripes represent exons, and pink stripes represent non-coding sequences (CNSs). The graph uses a critical value of 50 % identity. The Y axis represents the identity percentage in the range of 50-100 %. Fig. S5. Codon distribution map in the Dactylis mt genome. Red indicates a high relative synonymous codon usage (RSCU) value and green indicates a low RSCU value. Hierarchical clustering (average linkage method) was performed for the codon patterns (x-axis). Fig. S6. The base sequence dot-plot diagram of Dactylis aschersoniana, Dactylis glomerata and other two species. [file 12864_2024_10145_MOESM1_ESM.pdf]

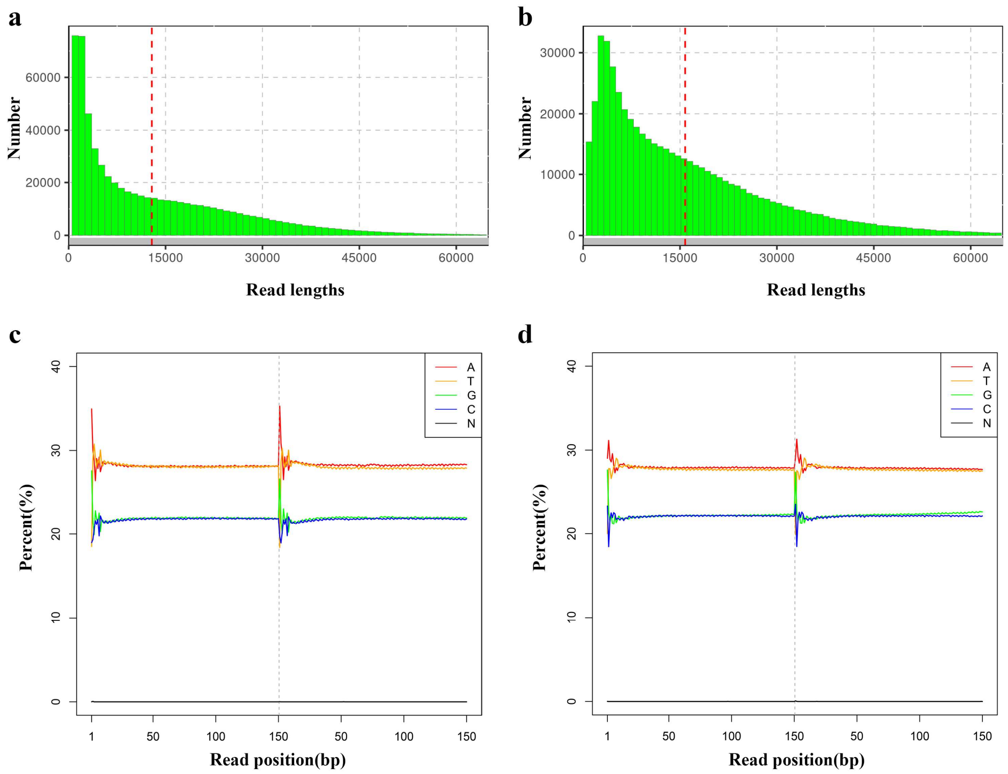

Fig. S1 The information of sequencing data. a and b, The raw data of the third-generation sequencing read length distribution of *Dactylis aschersoniana* and *Dactylis glomerata*, respectively; c and d, The A/T/G/C content distribution statistics of *Dactylis glomerata* and *Dactylis aschersoniana*, respectively.

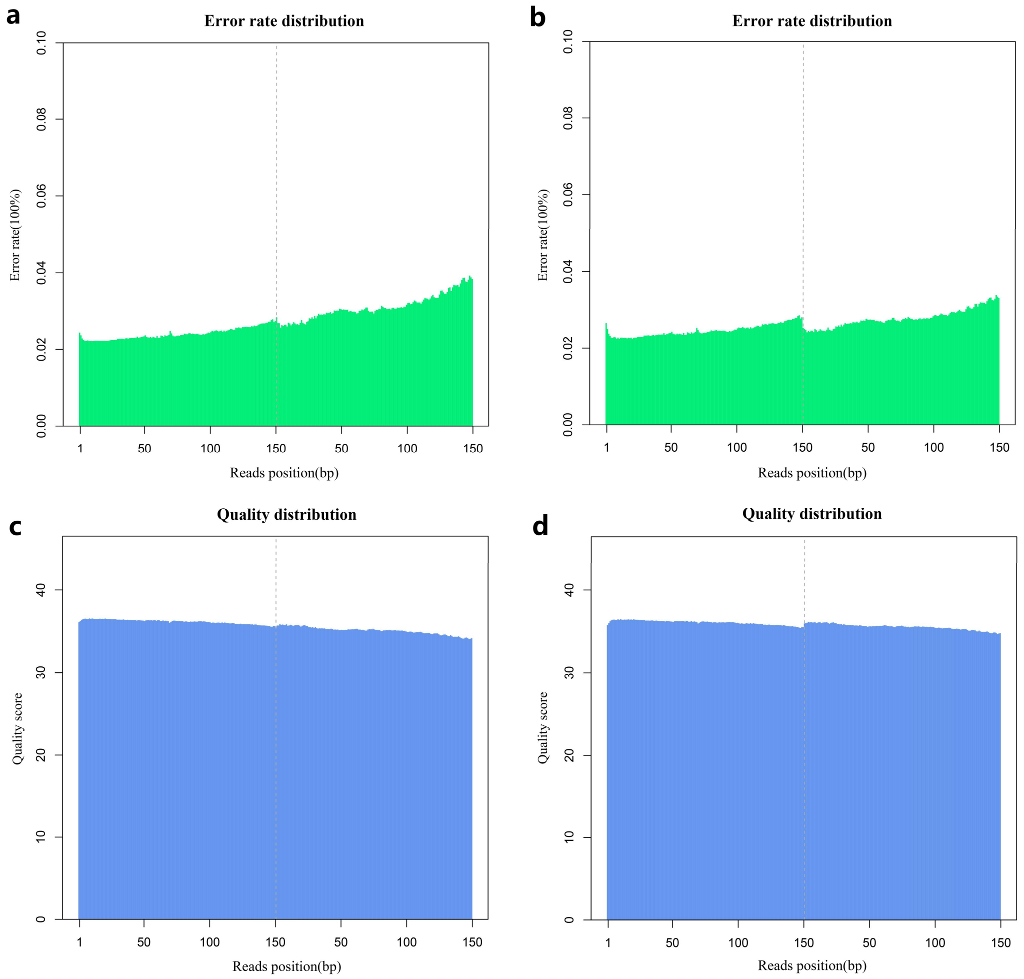

Fig. S2 Base error rate and quality distribution. a and b, The error rate distribution of *Dactylis aschersoniana* and *Dactylis glomerata*, respectively; c and d are quality distribution of *Dactylis aschersoniana* and *Dactylis glomerata*, respectively.

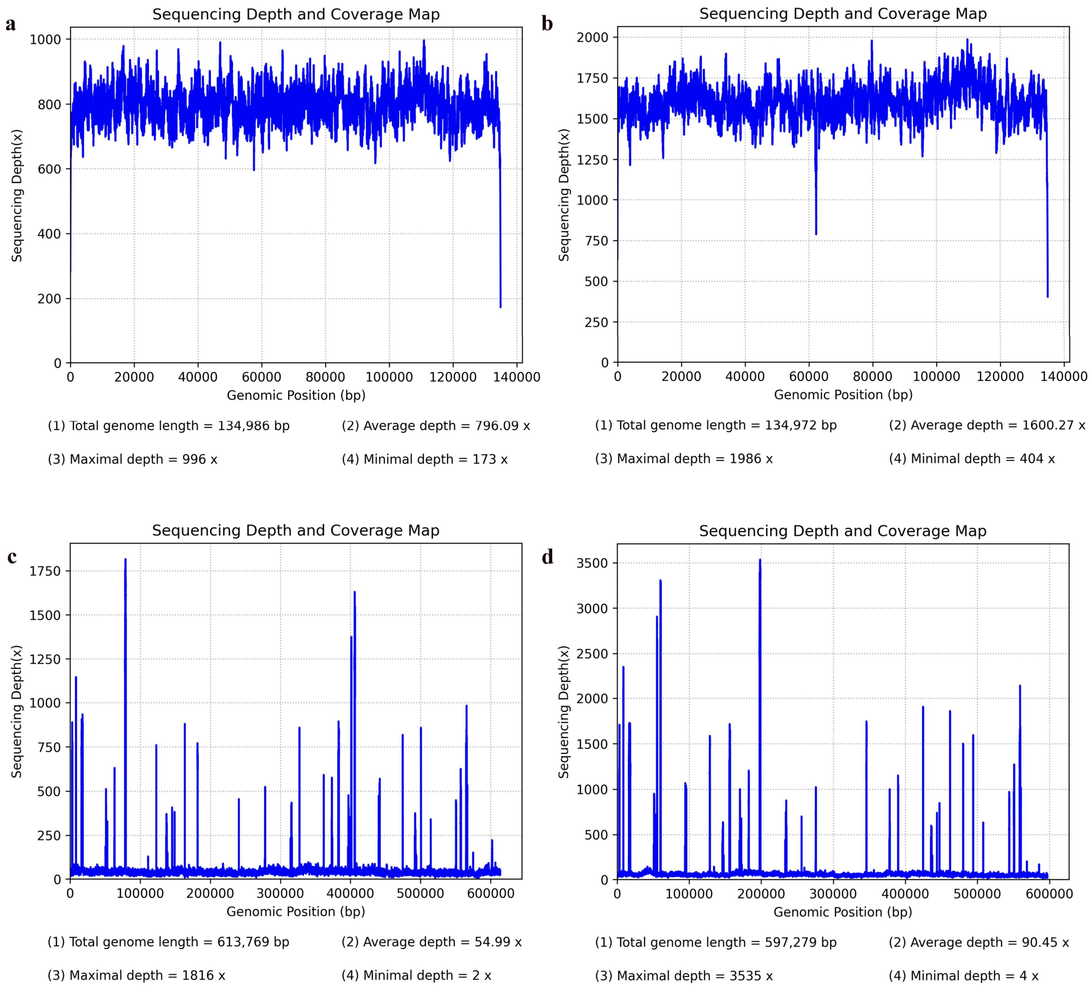

Fig. S3 Sequencing depth and coverage map of chloroplast and mitochondrial genomes. a and b represent the sequencing depth of coverage map from the chloroplast genomes of *Dactylis aschersoniana* and *Dactylis glomerata*. c and d represent the sequencing depth of coverage map from the mitochondrial genomes of *Dactylis aschersoniana* and *Dactylis glomerata.*

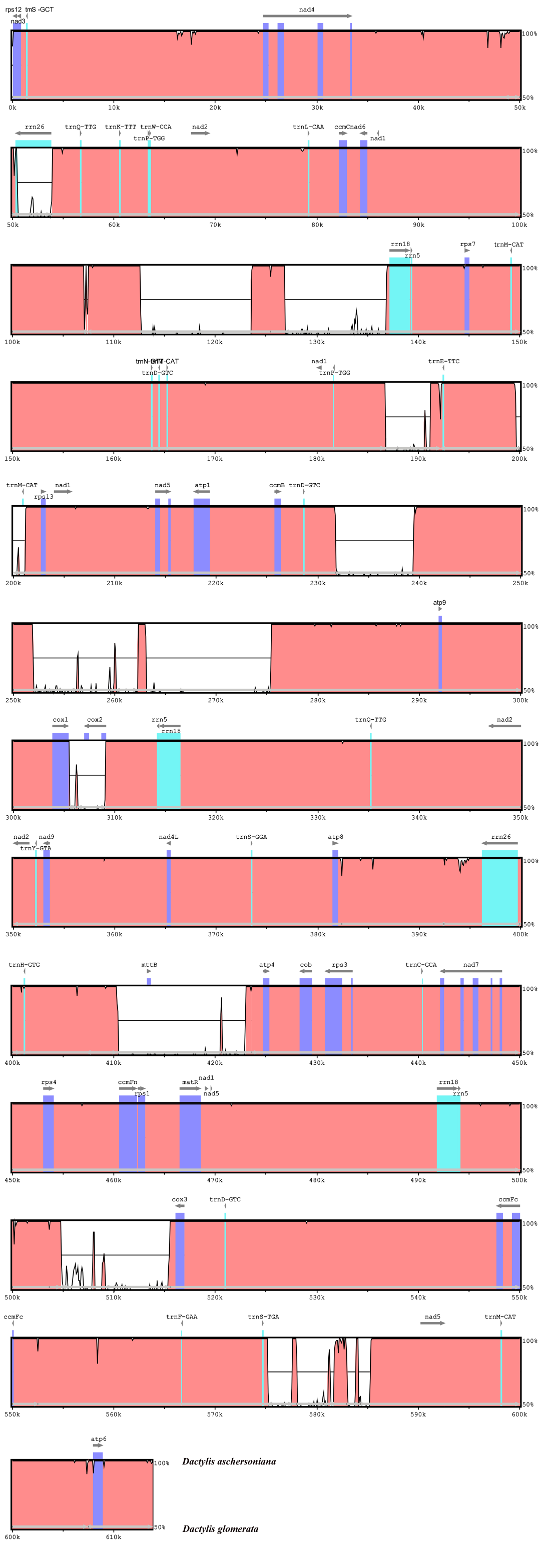

Fig. S4 The sequence identity maps of two *Dactylis* mitochondrial genomes. The gray arrow above the alignment indicates the direction of the gene. Blue stripes represent exons, and pink stripes represent non-coding sequences (CNSs). The graph uses a critical value of 50 % identity. The Y axis represents the identity percentage in the range of 50-100 %.

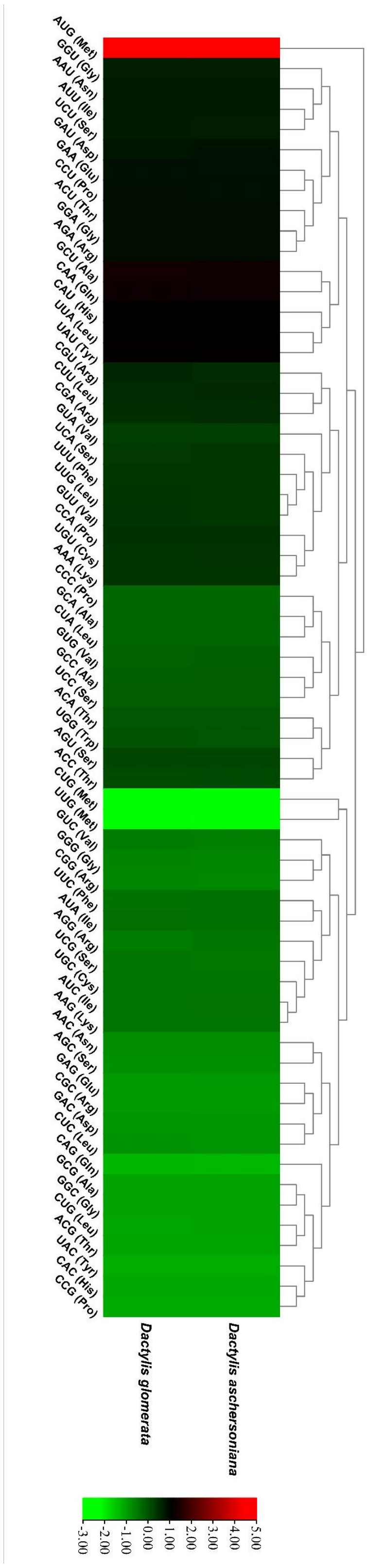

Fig. S5 Codon distribution map in the Dactylis mt genome. Red indicates a high relative synonymous codon usage (RSCU) value and green indicates a low RSCU value. Hierarchical clustering (average linkage method) was performed for the codon patterns (x-axis).

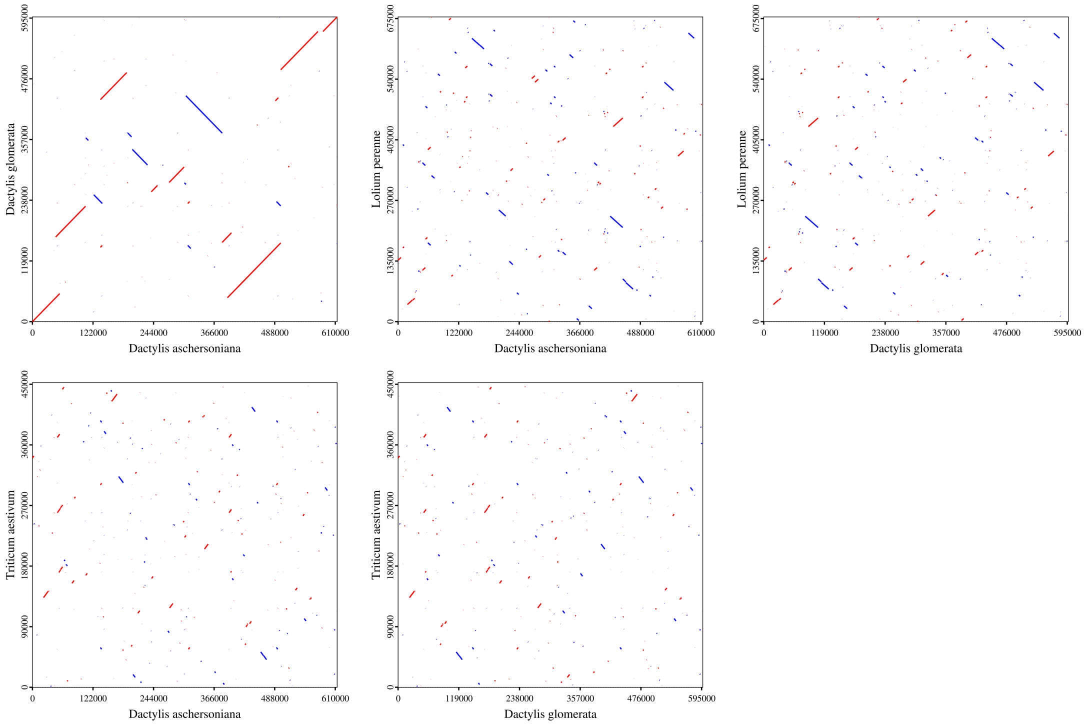

Fig. S6 The base sequence dot-plot diagram of *Dactylis aschersoniana*, *Dactylis glomerata* and other two species.
